# Proteomic screening identifies calreticulin as a miR-27a direct target repressing MHC class I cell surface exposure in colorectal cancer

**DOI:** 10.1038/cddis.2016.28

**Published:** 2016-02-25

**Authors:** T Colangelo, G Polcaro, P Ziccardi, B Pucci, L Muccillo, M Galgani, A Fucci, M R Milone, A Budillon, M Santopaolo, C Votino, M Pancione, A Piepoli, G Mazzoccoli, M Binaschi, M Bigioni, C A Maggi, M Fassan, C Laudanna, G Matarese, L Sabatino, V Colantuoni

**Affiliations:** 1 Department of Sciences and Technologies, University of Sannio, Benevento, Italy; 2 Centro Ricerche Oncologiche Mercogliano, Istituto Nazionale Tumori Fondazione G. Pascale—IRCCS, Mercogliano (AV), Italy; 3 Istituto di Endocrinologia e Oncologia Sperimentale, Consiglio Nazionale delle Ricerche (IEOS-CNR), Napoli, Italy; 4 Dipartimento di Medicina Molecolare e Biotecnologie Mediche, Università di Napoli ‘Federico II', Napoli, Italy; 5 Division of Epidemiology and Health Statistics, IRCCS—‘Casa Sollievo della Sofferenza' Hospital, San Giovanni Rotondo (FG), Italy; 6 Division of Internal Medicine and Chronobiology Unit, IRCCS—‘Casa Sollievo della Sofferenza' Hospital, San Giovanni Rotondo (FG), Italy; 7 Department of Pharmacology, Menarini Ricerche, Pomezia (RM), Italy; 8 Menarini Ricerche, Florence, Italy; 9 Department of Pathology and Diagnostic, ARC-NET Research Centre, University of Verona, Verona, Italy; 10 Department of Medicine (DIMED), Surgical Pathology Unit, University of Padua, Padua, Italy; 11 Department of Experimental and Clinical Medicine, Laboratory of Molecular Oncology, Magna Græcia University of Catanzaro, Catanzaro, Italy

## Abstract

Impairment of the immune response and aberrant expression of microRNAs are emerging hallmarks of tumour initiation/progression, in addition to driver gene mutations and epigenetic modifications. We performed a preliminary survey of independent adenoma and colorectal cancer (CRC) miRnoma data sets and, among the most dysregulated miRNAs, we selected miR-27a and disclosed that it is already upregulated in adenoma and further increases during the evolution to adenocarcinoma. To identify novel genes and pathways regulated by this miRNA, we employed a differential 2DE-DIGE proteome analysis. We showed that miR-27a modulates a group of proteins involved in MHC class I cell surface exposure and, mechanistically, demonstrated that calreticulin is a miR-27a direct target responsible for most downstream effects in epistasis experiments. *In vitro* miR-27a affected cell proliferation and angiogenesis; mouse xenografts of human CRC cell lines expressing different miR-27a levels confirmed the protein variations and recapitulated the cell growth and apoptosis effects. *In vivo* miR-27a inversely correlated with MHC class I molecules and calreticulin expression, CD8^+^ T cells infiltration and cytotoxic activity (LAMP-1 exposure and perforin release). Tumours with high miR-27a, low calreticulin and CD8^+^ T cells' infiltration were associated with distant metastasis and poor prognosis. Our data demonstrate that miR-27a acts as an oncomiRNA, represses MHC class I expression through calreticulin downregulation and affects tumour progression. These results may pave the way for better diagnosis, patient stratification and novel therapeutic approaches.

Colorectal cancer (CRC) is the third major cause of cancer worldwide.^1, 2^ In addition to gene mutations and epigenetic modifications, impairment of the immune response and dysregulation of microRNAs are emerging hallmarks of tumour initiation/progression.^3, 4, 5^ Tumour cells elicit a native and adaptive immune response mediated by different cell types aimed to eradicate the tumour.^6^ Antigen processing and presentation by major histocompatibility complex (MHC) class I molecules is a critical event to mount a specific antitumour response.^7^ Class I antigen peptides are originated from proteasome-mediated degradation of intracellular proteins and transported to the endoplasmic reticulum through the transporters associated with antigen-processing proteins (TAP1 and 2). Here they are loaded onto the peptide-loading complex (PLC) formed by GRP78, calnexin and tapasin on which calreticulin and ERp57 are recruited to interact with MHC class I molecules.^8, 9^ Once loaded with ‘optimal' antigens, these latter molecules dissociate from the PLC and translocate to the cell surface where they are recognized by cells of the immune system, contributing to immune surveillance.^9, 10, 11^

Defects of MHC class I antigen presentation occur at high frequency in solid tumours and is a feature of tumour immune evasion that renders cancer cells invisible to cytotoxic T cell. A selective loss or reduced level of MHC class I is generally associated with disease progression and reduced patient survival. Specifically, downregulation of MHC class I is detected in >74% of colorectal tumours and associated with a significantly shorter mean disease-specific survival compared with MHC-class-I-high-expressing tumours.^6, 12^

microRNAs dysregulation is a common feature of human malignancies as they act as oncomiRNAs or tumour-suppressor miRNAs.^4, 5, 13^ The contribution of miRNAs to antitumour immune response is still undefined and is a topic under intense investigation. In this study, we performed a preliminary survey of independent adenoma and CRC miRnoma data sets and, among the most dysregulated miRNAs, we selected miR-27a and showed that it is already upregulated in adenoma and further increases during the evolution to adenocarcinoma. Subsequently, using a proteomic approach, we identified a series of new proteins modulated by miR-27a that are involved in MHC class I cell surface exposure. In gain- and loss-of-function experiments, we provide evidence that miR-27a impairs this pathway directly targeting calreticulin. Furthermore, miR-27a greatly affects cell proliferation and angiogenesis *in vitro* and in mouse xenografts where these results are recapitulated. Consistently, miR-27a is overexpressed in a large proportion of human sporadic CRCs, inversely correlates with MHC class I molecules and calreticulin and CD8^+^ T cells' infiltration and activity. The combination of high miR-27a/low calreticulin is associated with distant metastasis and worse outcome.

## Results

### miR-27a is upregulated in human adenoma and CRC

**Figure 1:**
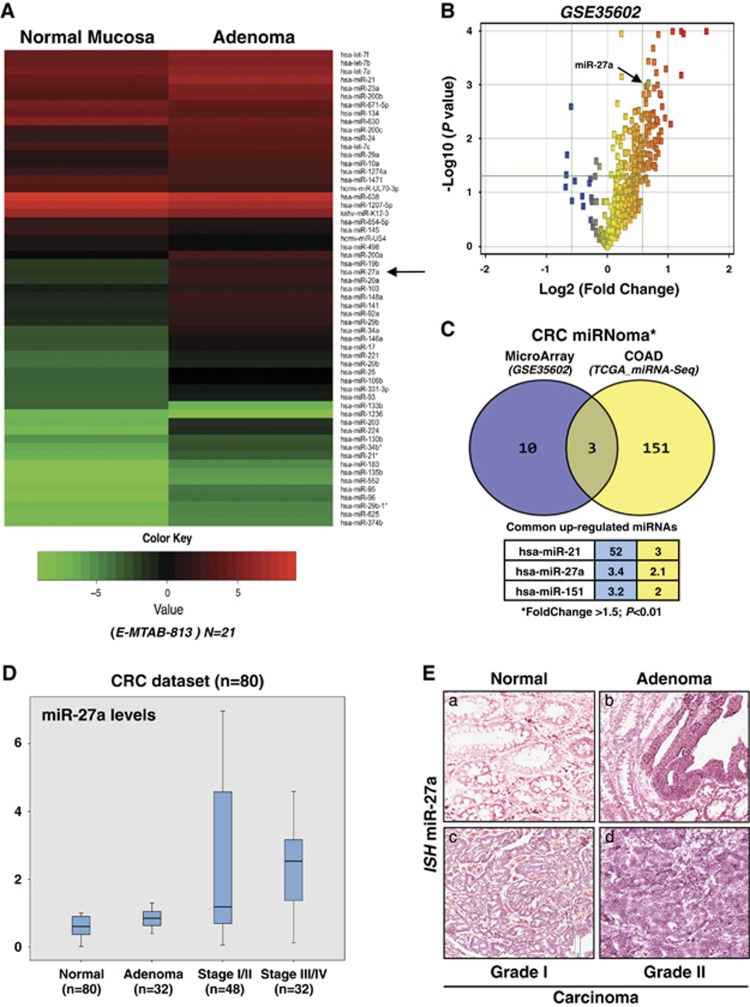
mir-27a upregulation is recognized *in silico* and confirmed in adenoma and CRC tissue analysis. (**A**) The heatmap shows the differentially expressed microRNA in an adenoma tissue series (*n*=21) with respect to normal mucosa (E-MTAB-813).^14^ The red arrow indicates miR-27a high levels in adenoma samples. (**B**) The differentially expressed miRNAs in 13 epithelial CRC samples and four normal tissues (GSE35602) are visualized in the Volcano plot (red squares; fold change >2, *P*≤0.05);^15^ miR-27a (green square) is arrowed. Green lines indicate the fold change and *P*-value thresholds. (**C**) The Venn diagram shows the number of differentially expressed miRNAs identified in independent and publicly available CRC data sets analysed by microarray (blue; GSE35602)^15^ or RNA-seq (yellow; TCGA COAD data set).^16^ Only the shared miRNAs and their relative expression levels are indicated in the table below the diagram. Fold change >1.5; *P*<0.01. (**D**) The box plot depicts miR-27a levels assessed by quantitative reverse transcriptase-PCR in our normal tissues, adenomas (*n*=32) and CRC samples (*n*=80) classified according to tumour stages (stages I–II, *n*=48; stages III–IV, *n*=32). (**E**) Representative *in situ* hybridization (ISH) of miR-27a at different stages of colorectal tumourigenesis (original magnifications × 10 and × 5)

We preliminarily analysed a publicly available adenoma data set for miRNA expression profile (E-MTAB-813);^14^ the heatmap reports the list of those that are upregulated or downregulated. We also analysed two independent CRC miRnoma data sets (GSE35602_microarray and TCGA_miRNA-seq)^15, 16^ and, upon intersecting the results as shown in the Venn diagram, identified three common upregulated miRNAs (Figures 1A–C). We selected miR-27a for further analysis and assessed its expression in our series of adenomas (*n*=32) and sporadic CRCs (*n*=80) by quantitative RT-PCR analysis. miR-27a was already elevated in about 60% of adenomas and further increased during tumour progression (stages I–II, *n*=48; stages III–IV, *n*=32), suggesting that its aberrant expression is an early event in colon tumourigenesis (Figure 1D). These data were confirmed by *in situ* hybridization that showed high levels in adenomas that increased in differentiated and even more in undifferentiated tumour samples (Figure 1E).

### Identification of novel genes and pathways regulated by miR-27a by differential proteome analysis

**Figure 2:**
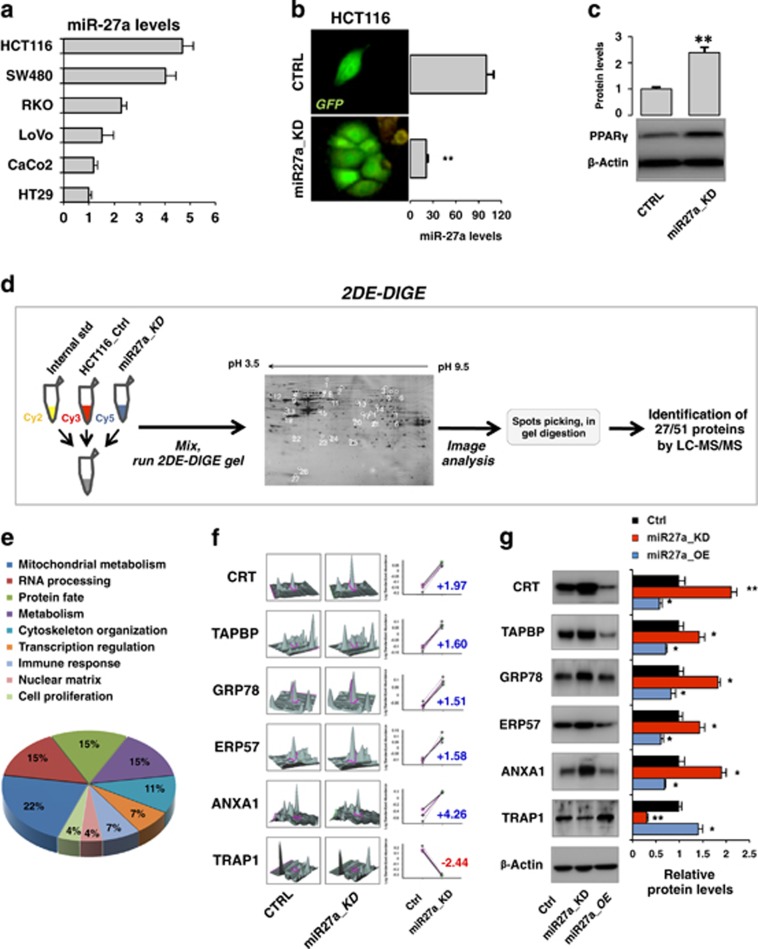
Proteomic (2DE-DIGE) profiling identifies novel proteins and pathways modulated by miR-27a in CRC cell lines. (**a**) miR-27a expression was analysed by quantitative reverse transcriptase-PCR in the indicated CRC cell lines. The histogram reports the fold change with respect to HT29 cells taken as control. U6 RNA was used as calibrator. (**b**) HCT116 cells were stably transfected with the miR-Zip-27a vector, carrying the miR-27a antisense and the GFP reporter gene, or with the empty vector as control. Cells were isolated for GFP expression and for the miR-27a low-expressing clones (miR27a_KD); **P≤0.01 (two-tailed Student's *t*-test). (**d**) Schematic representation of the 2DE-DIGE/MS procedure from sample preparation and labelling to gel analysis and protein identification. A representative image of a gel loaded with extracts from HCT116 CTRL and miR27a_KD cells is illustrated: about 1200 protein spots were identified, quantified, normalized, and inter-gel matched. Proteins were recognized by fold change of the corresponding spots. (**e**) The proteins identified by the 2DE-DIGE were classified into nine functional classes, according to their biological activities, as depicted in the pie chart. (**f**) A three-dimensional view of the 2DE-DIGE quantification is illustrated along with the standard abundance of the spots of interest identified in the gels obtained from HCT116 CTRL and miR27a_KD cells extracts. (**g**) Western blotting analysis of the selected proteins identified as differentially expressed in the 2DE-DIGE/MS analysis in HCT116 CRTL, miR27a_KD and miR27a_OE cells (HCT116-derived cell line that overexpresses an exogenous miR-27a). The relative protein fold change, obtained by densitometric analysis and normalization to *β*-Actin, is reported in the corresponding histogram. *P≤0.05; **P≤0.01 (two-tailed Student's *t*-test). Data are representative of three independent experiments and error bars represent S.D. of technical replicates (mean±S.D.) in panels (**a**), (**b**), (**c**) and (**g**)

To identify novel proteins and pathways modulated by miR-27a, we employed a proteomic approach. We first assessed its expression in a series of CRC-derived cell lines and selected HCT116 among the overexpressing ones (Figure 2a). A plasmid vector carrying a short hairpin anti-miR-27a RNA (shRNA) and the GFP (green fluorescence protein) cassette was stably transfected into HCT116 cells (CTRL); a cell clone, hereafter defined miR27a_KD, was chosen for further studies (Figure 2b). The efficacy of the silencing was established by assessing diminution of miR-27a and increase of validated targets (*PPARG*, *ZBTB10* and *FBXW7*) (Figure 2c and Supplementary Figure S1A).^17, 18, 19, 20^ We also transfected HCT116 cells with a plasmid carrying the miR-27a mimic and, among the overexpressing clones, we selected one hereafter named miR27a_OE. Extracts from CTRL and miR27a_KD cells were analysed by comparative proteomic 2DE-DIGE showing distinct expression profiles and a consistent experimental reproducibility (Supplementary Figure S1B, Supplementary Table S1). Out of the 51 differentially expressed spots, 27 were identified by LC-MS/MS, correlated with the corresponding spots and classified into 9 functional classes according to their biological activities (Figures 2d and e, Supplementary Table S2). Quantification of the selected spots is shown in a three-dimensional view along with the corresponding standard abundance in the two different cell lines (Figure 2f). Validation by western blotting analysis revealed an increase of calreticulin (CRT), tapasin (TAPBP), GRP78, ERp57 and annexin A1 (ANXA1) and reduction of TRAP1 in miR27a_KD with respect to CTRL cells; the inverse results were obtained in miR27a_OE (Figure 2g).

### miR-27a downmodulates MHC class I cell surface exposure

**Figure 3:**
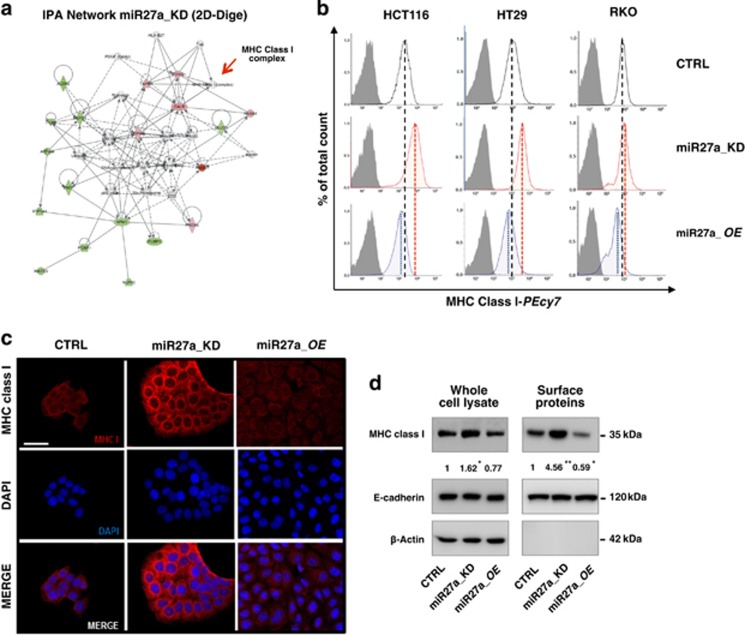
MHC class I cell surface exposure is downregulated by miR-27a in CRC cells. (**a**) The drawing shows the most enriched networks generated from the list of differentially expressed (DE) proteins (red elements=upregulated proteins; green elements=downregulated proteins) after miR-27a silencing using IPA. (**b**) Flow cytometry analysis reveals different MHC class I molecules cell surface exposure in HCT116, RKO, HT29 and their derivative clones miR27a_KD or miR27a_OE. (**c**) Immunofluorescence staining using an antibody against human MHC class I molecules in HCT116 CTRL and their derivative clones miR27a_KD or miR27a_OE (scale bar, 50 *μ*m). (**d**) Enrichment of MHC class I molecules in the isolated plasma membrane fraction. Positivity for E-cadherin, a membrane protein, and negativity for *β*-Actin, a cytosolic protein, proved that the identified proteins were truly integral membrane components. The relative fold change, obtained by densitometric analysis and normalization to E-cadherin, is reported below the bands. *P≤0.05; **P≤0.01 (two-tailed Student's *t*-test). Data are representative of three independent experiments and error bars represent S.D. of technical replicates (mean±S.D.)

To recognize the pathways in which the identified proteins are involved, we queried the Ingenuity Pathway Analysis (IPA) algorithm:^21^ antigen presentation, immunological, and inflammatory pathways were the most enriched ones (Figure 3a, Supplementary Figure S1C). Accordingly, we assessed cell-surface exposure of MHC class I molecules in three different cell lines (HCT116, HT29 and RKO) and their derivative clones with either a silenced or overexpressed miR-27a. In flow cytometry, miR27a_KD cells displayed more MHC class I proteins on the surface than the parental CTRL cells, whereas the miR27a_OE cells displayed less proteins (Figure 3b). Similarly, in immunofluorescence staining, the specific antibody recognized a low amount of MHC class I proteins on the membrane of non-permeabilized HCT116 CTRL cells that remarkably increased on the surface of miR27a_KD, whereas diminished in miR27a_OE cells (Figure 3c). The specificity of the staining was validated by counterstaining the nuclei with DAPI: by merging, the two stainings remained separate as they mark different subcellular compartment. To have a more quantitative assessment of the proteins exposed on the cell surface, we set up a procedure to selectively isolate plasma membrane proteins and evaluate those included. The miR27a_KD cells membrane fraction contained at least four times more MHC class I molecules than CTRL and even more than miR27a_OE cells. That the bands corresponded to real membrane proteins was confirmed by challenging the same fraction with E-cadherin, an integral membrane protein, as a positive control, and with *β*-Actin, a cytosolic protein as a negative control (Figure 3d). All together these data demonstrate that the surface expression of MHC class I molecules is downregulated by miR-27a.

### miR-27a directly targets calreticulin affecting MHC class I exposure

**Figure 4:**
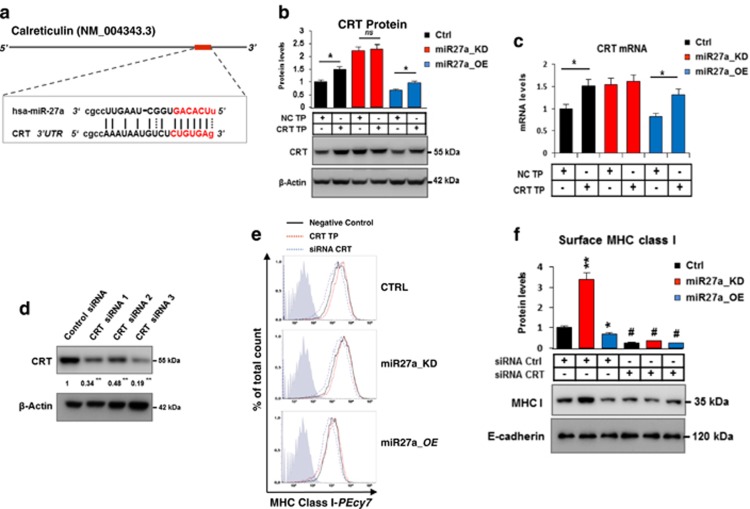
Calreticulin is a miR-27a target and affects MHC class I cell surface exposure. (**a**) Schematic representation of human calreticulin (CRT) mRNA: the highly conserved miR-27a-binding site located in the 3′UTR is illustrated in red. (**b**) Immunoblot analysis of CRT levels in HCT116 CTRL and derivative clones miR27a_KD or miR27a_OE cells after transfection with the specific target protector (CRT-TP) or negative control (NC-TP) for 72 h. *β*-Actin was used as a loading control. *P≤0.05, ns=non significant. (**c**) CRT mRNA qRT-PCR analysis of HCT116 CTRL and derivative clones miR27a_KD or miR27a_OE cells transfected with the specific CRT-TP or negative control (NC-TP) for 72 h. *P≤0.05 (Student's *t*-test). (**d**) CRT immunoblot analysis of HCT116 CTRL and derivative clones miR27a_KD or miR27a_OE cells transfected with three specific CRT siRNAs or Control siRNA for 72 h. Densitometric analysis is reported below the bands. **P≤0.01 (Student's *t*-test). (**e**) Flow cytometry assessment of cell surface MHC class I molecules in the same cells as in (**b**) after transfection with CRT-TP or CRT-siRNAs or relative negative controls for 72 h. (**f**) Immunoblot analysis of MHC class I molecules present in the plasma membrane fraction of HCT116 CTRL cells and their derivative clones miR27a_KD or miR27a_OE transfected with the CRT-siRNAs or scrambled siRNA control (Ctrl-siRNA). Data normalized to E-cadherin are shown in the histograms. *P≤0.05, **P≤0.01 versus HCT116 CTRL cells; ^#^*P*≤0.05 versus Ctrl-siRNAs transfected cells

To identify a direct target of miR-27a among the differentially expressed proteins, we inquired several algorithms and predicted calreticulin as a putative target owing to a conserved seed recognition sequence in the 3′UTR of the corresponding mRNA (Figure 4a).^22^ A miScript Target Protector (TP) was designed against this recognition sequence to selectively prevent the binding of miR-27a to the corresponding mRNA, without interfering with the action of the miRNA on other targets. Upon TP transfection, calreticulin increased in HCT116 and miR27a_OE more than miR27a_KD cells (Figure 4b). Interestingly, also the cognate mRNA increased, likely owing to a stabilization effect, contributing to the protein elevation detected in HCT116 and miR27a_OE cells (Figure 4c). Transfection of three independent siRNAs against calreticulin mRNA in HCT116, miR27a_KD and miR27a_OE cells successfully silenced the protein, with siRNA#3 as the most efficient (Figure 4d). To determine the impact of the miR-27a/calreticulin axis on MHC class I molecules surface exposure, we transfected calreticulin TP and siRNAs in the three cell lines. In flow cytometry, TP produced no significant variations, whereas the siRNAs, especially siRNA#3, increased MHC class I cell membrane display by 50% in miR27a_KD and 25–30% in CTRL and miR27a_OE cells (Figure 4e), in line with the total content of the isolated plasma membrane fraction (Figure 4f). Calreticulin, thus, is a direct target of miR-27a and mediates the effects on MHC class I exposure.

### Mouse xenografts recapitulate miR-27a effects on the proteomic profile and cell growth

**Figure 5:**
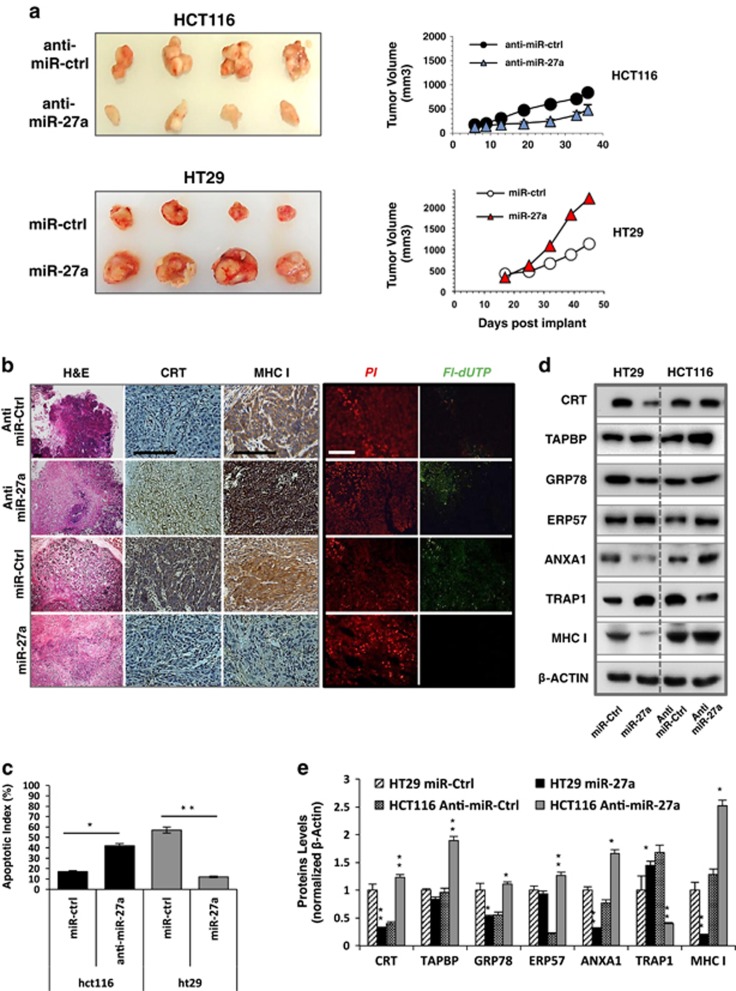
miR-27a acts as an oncogenic miRNA in mouse xenografts. (**a**) Representative photographs of the xenograft tumour masses excised from each experimental group. miR-27a inhibitor (anti-miR-27a; *n*=5) and scrambled control (miR-Ctrl; *n*=5) in HCT116 cells or miR-27a mimic (miR-27a; *n*=5) and scrambled control (miR-Ctrl; *n*=5) in HT29 cells were intratumourally injected every 7 days for four cycles starting from the day in which tumours reached the volume of 200 mm^3^. The growth curve of the tumours is reported for both types of xenografts. (**b**) Representative photomicrographs of haematoxylin and eosin staining, calreticulin and MHC class I IHC analysis and TUNEL assay, respectively, performed on paraffin-embedded sections of the same tissues; scale bars, 200 *μ*m. (**c**) The histogram shows the apoptotic index as determined by TUNEL assay. (**d**) Western blotting analysis of the selected proteins gene identified by 2DE-DIGE performed on total protein extracts of mouse xenografts and (**e**) their relative quantification. Data are represented as means±S.D. from at least two independently treated tumours; *P≤0.05; **P≤0.01 (Student's *t*-test)

To investigate the modulation of miR-27a on the proteins identified by 2DE-DIGE *in vitro*, we generated mouse xenografts. HCT116 and HT29, representative of miR-27a-overexpressing or -downexpressing cells, respectively, were subcutaneously transplanted into nude mice. After 2 weeks, a miR-27a inhibitor or scrambled controls were intratumourally injected every 7 days for four times in HCT116-derived tumours. Alternatively, a miR-27a mimic or scrambled controls were injected in HT29-derived tumours. At day 36 from transplantation, tumour masses were measured, excised and analysed. The size of HCT116-derived malignancies was remarkably larger (>50%) than those injected with the miR-27a antisense. Also, the tumours obtained upon injection of a miR-27a mimic into HT29 cell-derived masses were >50% larger than those from the parental cells (Figure 5a). The specificity and efficacy of miR-27a inhibition or overexpression was verified by qRT-PCR on total RNAs extracted from the two different types of tumours. In accordance with the size, Ki67 positivity was stronger in the high miR27a-expressing tumours than the lower ones, supporting a role of this miRNA in cell proliferation (Supplementary Figures S3A and B). By contrast, apoptosis was greatly reduced in the same tumours, whereas large areas of apoptosis were detected in those expressing low miR-27a by a terminal deoxynucleotidyl transferase (TdT)-mediated dUTP nick end-labelling test (TUNEL) assay (Figures 5b and c).

Calreticulin, ERp57, GRP78/BiP, Annexin1 and Tapaxin were downregulated in western blotting analysis of extracts from HCT116 tumours, whereas they were all upregulated in those with a silenced miR-27a. TRAP1 exhibited an inverse behaviour, in agreement with the 2DE-DIGE results *in vitro* (Figures 5d and e). The analysis of the same markers in HT29 tumours produced an opposite scenario, in line with the lower expression of miR-27a that was reversed upon injection of the corresponding mimic. No variations for all the indicated proteins were observed in the western blots of extracts from tumours injected with scrambled control with respect to HCT116 or HT29-derived tumours (data not shown).

MHC class I expression was monitored by immunohistochemistry (IHC). The signal detected on tissue sections from miR-27a antisense-injected tumours was stronger than from the scrambled injected or parental cell tumours. At a higher magnification, the staining was localized at the cell membrane, consistent with stimulation of MHC class I cell surface translocation upon miR-27a silencing. A more quantitative western blotting analysis on extracts from the same tumours confirmed in a mouse model that miR-27a silencing was associated with an overall increase of MHC class I proteins (Figures 5b, d and e). Consistently, calreticulin displayed a weak and mainly cytosolic positivity on sections of HCT116 tumours by IHC. The staining was, instead, remarkably localized as ‘patches' on cell membranes in tumours injected with a miR-27a antisense (Figure 5b). Western blotting analysis of extracts from the same tissues exhibited an overall increase of calreticulin only in those masses with a reduced miR-27a (Figures 5d and e). Collectively, mouse xenografts confirmed that miR-27a affects cell growth and apoptosis also in CRC and clearly showed that it negatively modulates a specific set of proteins identified *in vitro* that specifically contribute to MHC class I expression. These results definitely demonstrate that miR-27a is a CRC tumour-inducing factor acting as an oncomiRNA.

### miR-27a expression in CRC inversely correlates with MHC class I and calreticulin expression and with CD3^+^ and CD8^+^ T cells' infiltration/activation

**Figure 6:**
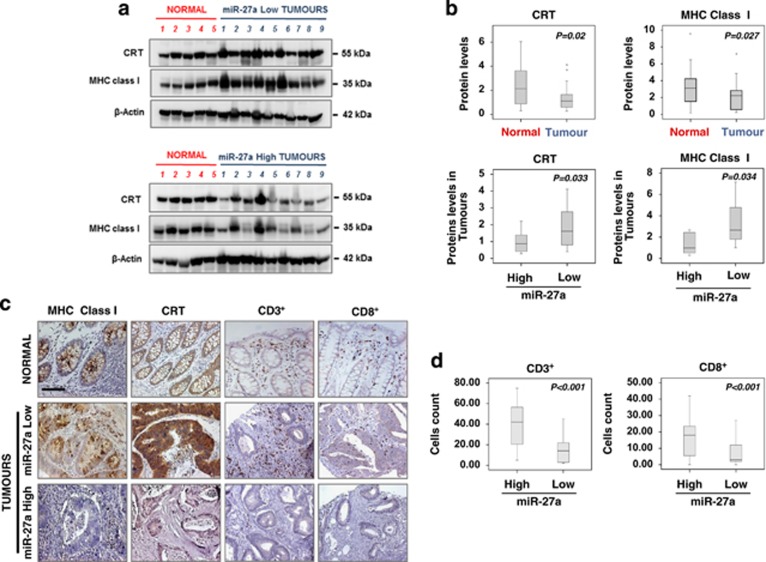
miR-27a upregulation in CRC samples correlates with reduced MHC class I and calreticulin expression and CD3^+^ and CD8^+^ T cells infiltration. (**a**) Western blotting analysis of CRT and MHC class I molecules in a representative group of normal tissues (*n*=5) and CRC samples (*n*=9) classified according to miR-27a expression; *β*-Actin was used as a loading control. (**b**) The box plots report CRT and MHC class I levels in normal *versus* tumour tissues (upper) and in tumour tissues according to miR-27a levels (lower). (**c**) IHC analysis of MHC class I, CRT, CD3^+^ and CD8^+^ T cells infiltrates in paraffin-embedded samples of normal and CRC specimens classified according to miR-27a levels (scale bar, 50 *μ*m). (**d**) Box plots report CD3^+^ and CD8^+^ T cells infiltration related to miR-27a levels. *P*-values were calculated by paired *t*-test in panels (**b**) and (**d**)

**Figure 7:**
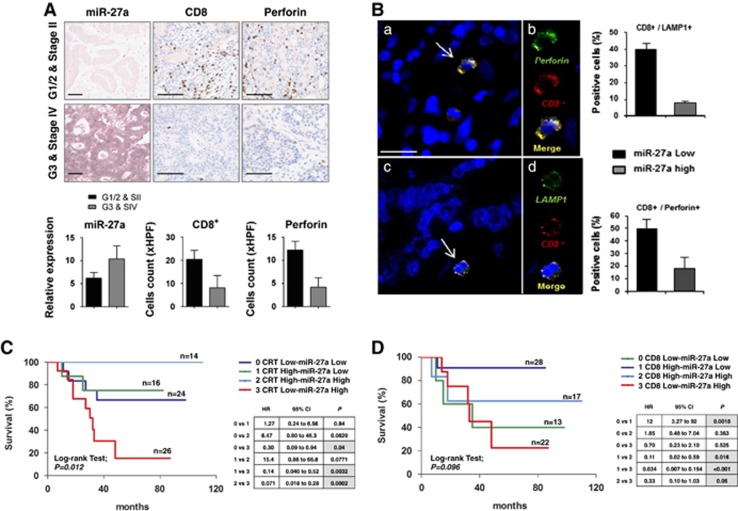
miR-27a is inversely associated with CD8^+^ T cells infiltration and activation affecting tumour aggressiveness and patients' survival. (**A**) IHC analysis of miR-27a, CD8^+^ T cells and perforin in paraffin-embedded samples of CRC specimens classified according to different tumour stages (stages I–II *versus* III–IV) (scale bar, 50 *μ*m). The histograms below report the quantification of CD8/perforin staining expressed as the mean number of positive cells in high-power fields (HPFs). (**B**) Immunofluorescence analysis of double-stained CD8^+^ (red) and LAMP1^+^ (green) or CD8^+^ and perforin^+^ cells (scale bars: 20 *μ*m in panels **a** and **b**, and 5 *μ*m in panels **b** and **d**). The histograms on the right show the percentage of double-positive CD8^+^/LAMP1^+^ and CD8^+^/perforin^+^ cells in tumour tissues according to miR-27a levels **P≤0.01 (two-tailed Student's *t*-test). (**C** and **D**) Kaplan–Meier survival analysis of CRC patients on the basis of (**C**) CRT/miR-27a and (**D**) CD8^+^/miR-27a combinations. Log-rank test; *P*=0.012, *P*=0.096. Hazard ratios analyses are reported in the boxes on the right side

To correlate the data obtained *in vitro* and in mouse xenografts with human CRCs, we performed quantitative western blotting analysis of some representative samples: high miR-27a-expressing CRCs displayed low MHC class I molecules and calreticulin (Figures 6a and b). Accordingly, IHC of tissue microarrays showed that high miR-27a-expressing tumours frequently displayed a weak or absent membrane staining for MHC class I molecules and calreticulin; the staining was stronger in low miR-27a-expressing tumours (Figure 6c). Furthermore, miR-27a inversely correlated with CD3^+^ and CD8^+^ T cells' infiltration and perforin positivity whose relative abundance was determined (Figures 6c and d). Perforin and LAMP-1 are two membrane proteins used as surrogate markers of CD8^+^ T cells' cytotoxic activity.^23^ Thus CD8^+^/perforin^+^ and CD8^+^/LAMP-1^+^ double-positive cells, detected by immunofluorescence on CRC specimens, were higher in low miR-27a-expressing tumours (Figures 7A and B). Altogether, these results suggest that miR-27a could impair T cells' infiltration, activation, proliferation and degranulation.^24^ Consistently, Kaplan and Meier analysis of patients' survival showed that low calreticulin expression (*P*<0.001) and CD3^+^ and CD8^+^ low infiltrates (*P*<0.001), taken alone, were significantly associated with a shorter overall survival, whereas high miR-27a showed only a trend (*P*=0.104; Supplementary Figures S4A and B). The low calreticulin/high miR-27a association (*n*=26) was the one with the worst outcome when these characteristics were combined; hazard ratios analysis of all possible associations identified calreticulin as a dominant variable that was even more discriminant when coupled with high miR-27a expression. When the CD8^+^ infiltrates were associated with miR-27a levels, the combination low CD8^+^/high miR-27a had the worse prognosis; hazard ratios analysis of all possible associations highlighted the presence of CD8^+^ infiltrates as a dominant variable (Figures 7C and D). High miR-27a/low calreticulin was also associated with the development of liver metastasis and CD3^+^/CD8^+^ T cells' infiltrates were reduced in metastases compared with matched primary tumours (Supplementary Figures S4C–E).

The biological relevance of these data was confirmed by two publicly available data sets.^15, 16^ miR-27a expression remarkably increased with tumour staging and inversely correlated with patients' overall survival, consistent with the results of our data set (Supplementary Figure S5A). Interestingly, whereas calreticulin mRNA was elevated in all data sets, the corresponding protein was reduced, a discrepancy explained by the posttranscriptional control mediated by miR-27a reported here (Supplementary Figures S6A and B; Figures 6a–c). miR-27a inversely associated also with CD3^+^ and CD8^+^ T cells' mRNAs from the early tumour stages and correlated with poor prognosis, supporting the results of our series (Supplementary Figures S6C–E). Collectively, miR-27a acts as an oncomiRNA from the early phases of colon tumourigenesis, impairs MHC class I and calreticulin expression, correlates with CD3^+^/CD8^+^ infiltration, development of distant metastases and poorer outcome likely affecting the host antitumour immune response *in vivo*.

## Discussion

Unveiling the full range of a microRNA's functions is a major task as they are involved in a vast array of biological processes. In addition, they simultaneously target many genes acting in different pathways, the interactions of which generate a network that can be different in distinct contexts and cell types.^4, 5, 13^ By a 2DE-DIGE proteomic approach, we identify a series of proteins modulated by miR-27a implicated in MHC class I expression. Specifically, miR-27a represses MHC class I surface exposure directly targeting calreticulin, a protein involved in the quality control of the assembly of this multi-subunit complex contributing to its stability and retrieval of suboptimally assembled MHC class I molecules.^7, 8, 9, 10, 11^ Mechanistically, calreticulin is a major downstream effector of miR-27a in repressing MHC class I surface exposure, a pivotal event in eliciting an efficient immune response and tumour eradication. Defects in this process are a common means for cancer cells to evade T cells' recognition.^25, 26^ The *in vivo* data support this notion: high miR-27a-expressing tumours inversely correlate with MHC class I expression in our CRC series. Although further studies are required to provide mechanistic insight into the link between miR-27a and MHC class I antigen presentation and, ultimately, CD8^+^ T cells' recognition and activation, our present data indicate that the miR-27a/calreticulin axis regulates MHC class I cell surface expression. miR-27a upregulation occurs from the very early phases of colorectal tumourigenesis and persists throughout the progression accounting for a more aggressive development. In line, CRCs expressing the combination high miR-27a/low calreticulin are associated with reduced CD3^+^/CD8^+^ T cells' infiltrates and cytotoxic activity, a more aggressive behaviour, metastatic spreading and worse outcome. The emerging scenario is that tumour and neighbouring cells, especially infiltrating immune cells, establish a specific microenvironment through an intricate network of crosstalks.^27, 28^ miR-27a is crucial in immune cells, as it inhibits DCs maturation and T cells' proliferation and activation, whereas induces M2b and M2c macrophage subtypes maturation.^29, 30, 31^ Here we provide evidence that miR-27a has a key role also in tumour cells by repressing MHC class I cell surface exposure. miR-27a appears to be a multifaceted signaling molecule that may influence tumour cells–host immunological interactions likely disabling components of the immune system that have been dispatched to eliminate them.

In conclusion, we demonstrate for the first time that miR-27a modulates MHC class I surface exposure by directly targeting calreticulin. Our data support that miR-27a has a critical role in colon tumourigenesis likely influencing the antitumour immune response. The identification of a miR- 27a–calreticulin regulatory axis open up avenues for the search of novel strategies aimed at ameliorating patient prognosis and improving the therapeutic response.

## Materials and Methods

### Cell culture

Human colon cancer cell lines HCT116, HT29, CaCo-2, LoVo, RKO and SW480 were purchased from American Type Culture Collection (ATCC, Rockville, MD, USA). All cell lines were maintained in Dulbecco's modified Eagle's or RPMI 1640 medium supplemented with 10% fetal bovine serum, 2 mM L-glutamine, penicillin and streptomycin. Cells were cultured in a humidified 37 °C incubator at 5% CO_2_. Human umbilical cord endothelial cells were purchased from ATTC and maintained in EGM BulletKit medium (Lonza, Allendale, NJ, USA).

### Oligonucleotides and plasmids transfection

Synthetic miR-27a mimic (Syn-hsa-miR-27a), miR-27a inhibitor (anti-hsa-miR-27a) or the appropriate scrambled controls (AllStar or mirScript Inhibitor-Negative Control) were purchased from Qiagen (Hilden, Germany). The miR-27a-antisense (MZIP27a-PA-1), the pre-miR-27a expression constructs (PMIRH27a-onlyPA-1) and scrambled control miRNAs (MZIP000-PA-1; PMIRH000PA-1) plasmids (System Biosciences, Mountain View, CA, USA) were transfected in the different CRC cell lines. microRNA functional studies were performed by inhibiting miRNA–mRNA target interactions either with a custom-designed calreticulin-miScript Target Protector or a negative control miScript Target Protector (MTP0075035; Qiagen). Detection of no variations in unrelated proteins validated TP specificity. A gene-specific package of three preselected siRNAs against calreticulin (Flexi Tube siRNA GS811) or a negative control siRNA (SI03650325) (Qiagen) was used in transient transfections. Functional assays, RNA and protein analyses were performed within 24/72 h from transfections. In each experiment, the extent of miR-27a silencing/overexpression and calreticulin silencing were assessed by qRT-PCR and western blotting analysis, respectively. Plasmids and oligonucleotides were transfected using Lipofectamine 2000 (Thermo Fisher, Waltham, MA, USA), HiPerFect Transfection Reagent (Qiagen) or RNAi Max (Thermo Fisher), respectively, according to the manufacturers' recommendations.

### mRNA/miRNA extraction and qRT-PCR analysis

Total RNA was extracted from cells and tissues using TRIzol (Thermo Fisher) and treated with DNase I. microRNAs were extracted using the Qiagen miRNeasy Mini Kit (Qiagen) according to the manufacturer's protocol. Assessment of RNA purity and quantity was performed as described.^32^ The sequences of the specific primers are reported in Supplementary Table S3.

### Western blotting analysis

Protein extracts from cell lines and tissues were prepared and analysed as previously reported.^32^ Antibodies to calreticulin (ab2907), TAPBP (ab140982), ERP57 (ab13506) and MHC class I (ab70328) were from Abcam (Cambridge, MA, USA); PPAR*γ* (sc-7273), TRAP1 (sc-9134), GRP78 (sc-13968), anti-mouse (sc-2031) and anti-rabbit (sc-2004) were from Santa Cruz Biotechnology (Dallas, TX, USA); ANXA1 (71–3400) from Thermo Fisher, *β*-Actin (F-3022) from Sigma-Aldrich (Milan, Italy); E-cadherin (BD 610405) from BD transduction (BD Biosciences, San Jose, CA, USA). To analyse surface proteins, we used an extraction method based on a published procedure. Positivity for E-cadherin, a plasma membrane protein, and negativity for *β*-Actin, a cytosolic protein, proved that the identified proteins were truly integral membrane components. Comassie blue staining was also used for assessing equivalent protein load. Proteins were then analysed by western blotting as previously reported.^32, 33^

### 2DE DIGE analysis

Differential proteome analysis on HCT116 CTRL and HCT116 miR27a_KD was performed as previously described by Milone *et al.*^34^ The proteomic experiments included: (a) protein preparation and labelling with DIGE dyes; (b) isoelectrofocusing (IEF); (c) image acquisition, analysis and processing; and (d) protein identification using LC-MS/MS. The experimental design using the three-dye approach is illustrated in Supplementary Table S1.

### Animal experiments

For xenograft generation, 20 × 10^6^ CRC-derived cells (HCT116 or HT29) were subcutaneously transplanted into the flank of 20 female athymic nude mice (6–8-weeks old; Charles River, Lecco, Italy). Mice were maintained according to the United Kingdom Coordinating Committee on Cancer Research guidelines, and tumour volumes, calculated as (tumour length × width2)/2, were monitored twice a week by caliper measurement.^35, 36^ Two weeks after transplantation, when tumours reached the volume of 200 mm^3^, mice were grouped (*N*=5/group) and intratumourally injected every 7 days for four times with anti–miR-27a (4 ng/mm^3^) for HCT116 or with miR-27a mimic (2 ng/mm^3^) for HT29 xenograft models. In both cases, the appropriate scrambled RNAs were used (indicated as anti-miR-Ctrl and miR-Ctrl, respectively). At day 36, tumour masses were measured, excised and further analysed; qRT-PCR was performed on RNA from xenografts to establish the efficiency of miR-27a inhibition/overexpression. This experiment was carried out in duplicate. No adverse or toxic effects were observed. All animal experiments were reviewed and approved by the Ethics Commission at Menarini Ricerche, according to the guidelines of the European Directive (2010/63/UE).

### TUNEL assay

To evaluate the apoptotic rate on xenograft tissues, paraffin-embedded tumour masses were analysed by the DeadEnd Fluorometric TUNEL System (Promega, Madison, WI, USA), according to the manufacturer's instructions. Image acquisition and data analyses were performed using a Carl Zeiss LSM700 laser-scanning microscope (Carl Zeiss, Jena, Germany).

### Immunofluorescence and IHC

Immunofluorescence staining was carried out on non-permeabilized HCT116 and derivative clones. Cells were plated on coverslips, fixed in para-formaldehyde (4% in PBS) at room temperature for 10 min, blocked in bovine serum albumin (3% in PBS) for 30 min before incubation with specific antibodies to MHC class I (1 : 100 dilution) for 1 h at room temperature. Subsequently, anti-mouse IgG-R secondary antibody (sc-2092, 1 : 1000 dilution) (Santa Cruz Biotechnology) was incubated for 1 h at room temperature. Coverslips were washed with PBS, stained with DAPI and, after three more washes in cold PBS, mounted in mowiol 4–88 (Merck-Millipore, Darmstadt, Germany) on glass slides. For double-immunofluorescence staining, after an initial block with 10% normal serum in PBS, tissue sections were incubated overnight at 4 °C with an unconjugated primary antibody specific for CD107a (also known as LAMP-1) FITC (ab25406) or a monoclonal antibody to perforin (Clone 5B10; Diagnostic Biosystems, Pleasanton, CA, USA) and CD8-PerCP-Cy5.5 fluorescence conjugated (BD Biosciences). For negative controls, primary antibodies were replaced with preimmune serum and IgG isotype controls. The fluorescence signals were observed and captured using a Carl Zeiss LSM700 laser-scanning microscope (Carl Zeiss). IHC and haematoxylin and eosin staining on human CRC tissues and mouse xenografted tissues were performed and evaluated as previously reported^32^ using antibodies against Ki67 (Dako, Milan, Italy), Calreticulin and MHC class I, CD3 (CD3-565-L-CE) and CD8 (CD8-295-CE) (Novocastra, Milan, Italy) and perforin (Diagnostic Biosystems). Image acquisition and analysis were performed on DM100 Leica Photosystem 40.106.206 (Leica, Milan, Italy).

### Flow cytometry

Flow cytometry was employed to detect MHC Class I molecules on the cell surface of HCT116, RKO and HT29 and their derivative clones miR27a_KD or miR27a_OE. Briefly, cells were plated, harvested and washed twice with PBS and incubated for 1 h in darkness at 4 °C with PE-Cy-7-labelled anti-MHC class I (561349) (BD Biosciences). Cells were then washed and resuspended in cold PBS for FACS analysis. All flow cytometry results were analysed with the FACSuite Software v.1.0.5.3841 (BD Biosciences).

### *In vitro* angiogenesis assay

The tube-formation assay was performed as previously described^32^ and is illustrated in Supplementary Figure S2A.

### *In situ* RNA hybridization

Formalin-fixed paraffin-embedded (FFPE) sections of tubular adenomas with low-grade/high-grade dysplasia or colorectal adenocarcinomas (G1, G2 and G3) were stained for miR-27a. Three cases per pathological sub-group were analysed. Three normal colonic mucosa biopsy samples, used as control, were obtained from patients who underwent colonoscopy for irritable bowel syndrome. miR-27a probe was labelled with 5-digoxigenin and synthesized by Exiqon (Vedbaek, Denmark). *In situ* hybridization was performed as described, with minor modifications.^37^ Negative controls included omission of the probe and the use of a scrambled LNA probe; U6 was used as positive control (Exiqon). Slides were counterstained in fast red solution. For miR-27a/CD8^+^/perforin expression analysis, primary sporadic CRCs were considered. Serial sections obtained from the original paraffin blocks were stained for miR-27a, CD8^+^ and perforin. Only cytoplasmic miR-27a intensity was retained for scoring, and miRNA expression was quantified analysing chromogen-specific intensity by Image J.^38^ IHC for CD8^+^ (Clone C8/144B; Dako) and perforin (Diagnostic Biosystem) were performed on the Benchmark LT automated system from Leica Microsystems Bondmax (Leica, Wetzlar, Germany) according to the manufacturer's specifications. The results of CD8^+^/perforin staining are expressed as the mean number of positive cells in high-power fields.

### Clinical samples

Paraffin-embedded and liquid nitrogen–frozen specimens from adenoma (*n*=32) and primary sporadic CRCs (*n*=80) (stages I–II, *n*=48; stages III–IV, *n*=32) were included in this study. Each sample was matched with the adjacent apparently normal mucosa (*n*=80) removed during the same surgery. Patients' familial history and tumour classification have been reported.^32^ Patients were followed up for a median of 89.79 months or until death. All patients gave informed consent for sample collection, and study protocols were approved by the Institutional Review Board of the Fatebenefratelli Hospital in accordance with the ethical guidelines of the Declaration of Helsinki.

### Independent data sets' analysis

The following independent, publicly available adenoma and CRC data sets, deposited in the Gene Expression Omnibus (GEO) as adenoma E-MTAB-813,^14^ (GEO) GSE35602 series (http://www.ncbi.nlm.nih.gov/geo website) and TCGA COAD series (https://tcga-data.nci.nih.gov/docs/publications/coadread_2012), respectively, were analysed for miR-27a expression in adenoma and CRC tissues to evaluate its prognostic significance. The adenoma data set consists of 21 patients while the data set GSE35602 counts on 59 RNA samples separately extracted from stroma and epithelium of 13 CRC tissues and four normal tissues.^15^ mRNA expression analysis was performed on Agilent-014850 Whole Human Genome Microarray 4x44K (Agilent Technologies, Santa Clara, CA, USA); for miRNA analysis, Agilent-019118 Human miRNA Microarray 2.0 G4470B was used. Robust multichip average normalization was performed using GeneSpring 11.5 (Agilent Technologies).^39^ The information from this data set was used to identify differentially expressed miRNAs having a fold change ≥2 and *P*<0.05, as determined by Welch *t*-test statistical analysis. We performed Volcano plot analysis to visualize differential expression. TCGA COAD data set^16^ consists of 224 colorectal tumours and normal pairs. Normalized Level 3 data were used for our analysis. IPA (Ingenuity Systems, (http://www.ingenuity.com website) was used for gene set enrichment analysis and gene network analysis.

### Statistics

All statistical analyses were made using Statistical Package from Social Science (SPSS; version 16.0) for Windows (SPSS Inc., Chicago, IL, USA) and R/Bioconductor (Seattle, WA, USA). Association between miRNA expression and tumour stage was assessed using Fisher exact test or Pearson χ2 test (where indicated). The Kaplan–Meier method was used to estimate survival; log-rank test was used to test differences between the survival curves; the hazard ratios were calculated by combining the variables at 95% confidence interval to correlate the chance of events. Data are reported as means±S.D., and mean values were compared using Student's *t*-test or Mann–Whitney test. Results were considered statistically significant when *P*≤0.05 was obtained.

## Abbreviations

CRC: colorectal cancer
PLC: peptide-loading complex
MHC: major histocompatibility complex
