# Supplementary material for: Proteomic screening identifies calreticulin as a miR-27a direct target repressing MHC class I cell surface exposure in colorectal cancer
Source: Cell Death Dis. 2016 Feb 25;7(2):e2120–. doi: 10.1038/cddis.2016.28 (PMC4849154; doi:10.1038/cddis.2016.28)
Supplement: Supplementary Information [file cddis201628x1.doc]

**Supplementary Information**

**Proteomic screening identifies calreticulin as a miR-27a direct target repressing MHC class I cell surface exposure in colorectal cancer**

Tommaso Colangelo^1^, Giovanna Polcaro^1^, Pamela Ziccardi^1^, Biagio Pucci^2^, Livio Muccillo^1^, Mario Galgani^3^, Alessandra Fucci^1#^, Maria Rita Milone^2^, Alfredo Budillon^2^, Marianna Santopaolo^4^, Carolina Votino^1^, Massimo Pancione^1^, Ada Piepoli^5^, Gianluigi Mazzoccoli^6^, Monica Binaschi^7^, Mario Bigioni^7^, Carlo Alberto Maggi^8^, Matteo Fassan^9^, Carmelo Laudanna^10^, Giuseppe Matarese^3,4^, Lina Sabatino^1§^ and Vittorio Colantuoni^1§^

**Table of contents**

**Supplementary Figures S1-6**

**Supplementary Tables S1-3**

**Supplementary Figure S1: Validation of known miR-27a targets and identification of novel pathways modulated in CRC cell lines.**

**(A)** qRT-PCR analysis of *PPARG, ZBTB10* and *FBXW7* mRNAs, known miR-27a targets, in HCT116 CTRL and miR27a_KD cells. ***P* ≤ 0.01 (Student’s t-test). **(B)** Principal component analysis (PCA) shows distinct expression profiles in HCT116 CTRL and miR27a_KD cells, indicating a consistent reproducibility among the biological triplicates of the experiments. **(C)** The histograms show the most significantly enriched pathways related to miR-27a variations (Red Arrow) obtained by IPA. The *x* and *y* axes indicate the degree and the statistical significance of enrichment, respectively.

**Supplementary Figure S2: miR-27a promotes CRC cells proliferation and angiogenesis.**

**(A)** Tube formation assay produced by HUVECs after incubation with the conditioned media (CM) from HCT116 CTRL, miR27a_KD and miR27a_OE cells. The histogram reports the mean number of tubes in at least 10 low-power fields; serum-free medium was used as negative control. **(B)** qRT-PCR analysis of proliferation markers (Cyclin D1, p21, p27 and PTEN) in the HCT116 and HT29 cells upon miR-27a silencing or overexpression. Data are represented as means ± s.d. of three independent experiments. * *P* ≤ 0.05; ** *P* ≤ 0.01 (Student’s t-test).

**Supplementary Figure S3: Experimental data supporting the results illustrated in the main figures.**

**(A)** qRT-PCR levels of miR-27a expression in xenograft tumours; *P*=0.104, *P*<0.001. **(B)** Immunohistochemistry of Ki67 in the same tumours as in **A** and the relative quantitation.

**Supplementary Figure S4:** **miR-27a, calreticulin and CD3^+^ and CD8^+^ levels correlate with CRC patients’ survival.**

**(A)** Kaplan-Meier survival analysis according to calreticulin and miR-27a expression. Log-rank Test; *P*=0.104, *P*<0.001. **(B)** Kaplan-Meier analysis according to CD3^+^ and CD8^+^ infiltrates. Log-rank Test; *P*<0.001. **(C)** The histograms show the correlation between the presence/absence of liver metastasis, miR-27a and calreticulin levels. *P*=0.029, *P*=0.034. *P* values are calculated by paired *t*-test. **(D)** The box plots show CD3^+^ and CD8^+^ infiltration levels in primary tumours vs metastasis. *P* values are calculated by paired *t*-test. **(E)** The box plots illustrate the association of CD8^+^ and CD3^+^ infiltrates with calreticulin levels. *P*<0.05. *P* values are calculated by paired *t*-test. All data refer to our dataset patients (n=80).

**Supplementary Figure S5:** **miR-27a expression survey of two independent and publicly available datasets.**

**(A)** The box plot on the left shows miR27-a levels in normal vs tumour tissues from the COAD dataset (GEO record GSE35602, 210 samples); the box-plot on the right shows the same analysis performed stratifying samples according to tumour stages (Stages I-IV) vs normal tissue. *P*<0.01. *P* values are calculated by paired *t*-test. Kaplan-Meier analysis according to miR-27a levels is shown. Log-rank Test: *P*=0.029.

**Supplementary Figure S6**: **Calreticulin, CD3^+^ and CD8^+^ mRNAs expression in normal vs tumour tissues and survival analysis in combination with miR-27a.**

Calreticulin mRNA levels in normal vs tumour tissues in **(A)** our dataset (*P*=0.003. *P* values are calculated by paired *t*-test) and in **(B)** the COAD dataset (GEO record GSE35602) (*P*=0.037. *P* values are calculated by paired *t*-test). **(C)** The box plots show the normalized expression of CD3^+^ and CD8^+^ in normal vs tumour tissues in our dataset. *P*=0.013, *P*=0.033. *P* values were calculated by paired *t*-test. Kaplan-Meier survival analysis in **(D)** takes into account the miR-27a/CD8^+^ combination (Log-rank Test; *P*=0.183) and in **(E)** the correlation between calreticulin and miR-27a levels in our dataset (Log-rank Test; *P*=0.151).

**Supplementary Table S1: Schematic representation of the reverse labeled Cy3 - Cy5 dyes for each biological replicate. Cy2 dye was used for internal standard labelling.**

**Supplementary Table S2: List of the proteins identified by LC-MS/MS from some spots present in the 2DE-DIGE gels, divided on the basis of their biological activities.**

**Supplementary Table S3: List of the sequences of the primers used.**
